# Stress and Displacement Dynamics in Surgically Assisted Rapid Maxillary Expansion: A Comprehensive Finite Element Analysis of Various Osteotomy Techniques

**DOI:** 10.3390/jcm14020449

**Published:** 2025-01-12

**Authors:** Müjde Gürsu, Mehmet Barış Şimşek

**Affiliations:** Department of Oral and Maxillofacial Surgery, Faculty of Dentistry, Gazi University, 06490 Ankara, Turkey; mbaris@gazi.edu.tr

**Keywords:** SARPE, finite element analysis, maxillary expansion, maxillary transverse deficiency, stress, displacement

## Abstract

**Objectives**: This study aimed to compare the effects of surgically assisted rapid palatal expansion (SARPE) techniques and their combinations on the stresses (von Mises, maximum principal, and minimum principal) and displacements that occur in the maxilla, facial bones, and maxillary teeth using three-dimensional finite element analysis (FEA). **Methods**: SARPE was simulated using seven different osteotomy techniques. The FEA models were simulated with a combination of various osteotomies, including midpalatal and lateral osteotomies, lateral osteotomy with a step, and separation of the pterygomaxillary junction. For each osteotomy variant, the instantaneous displacements and stresses resulting from forces applied by a 1 mm expansion of a tooth-borne appliance were evaluated. **Results:** Midpalatal osteotomy increased lateral displacement in the alveolar bone margins and intermaxillary suture while significantly reducing stresses around the intermaxillary suture. The addition of a pterygomaxillary osteotomy to the midpalatal and lateral osteotomies effectively reduced stresses in the posterior maxilla and cranial structures while enhancing lateral displacement. Although lateral osteotomy significantly reduced stresses in the midface, its effect on maxillary expansion was limited. Stepped lateral osteotomy had minimal effects on transverse displacement and stress reduction. **Conclusions**: Increasing the number of osteotomies reduced stress levels in the maxilla while enhancing lateral displacement. These results highlight the importance of selecting the most appropriate osteotomy technique to achieve optimal outcomes.

## 1. Introduction

Maxillary transverse deficiency is a common skeletal problem observed in the craniofacial region [1], and its etiology includes congenital, genetic, developmental, environmental, and iatrogenic factors [2,3]. Maxillary transverse deficiency is frequently associated with a unilateral or bilateral posterior crossbite, anterior crowding, a narrow and high palatal arch, and difficulties in nasal breathing and mastication [4]. In skeletally immature patients, slow or rapid maxillary expansion using orthodontic appliances to separate the midpalatal suture is a widely used treatment approach [5]. However, in adults, increased suture bone density and the completion of skeletal growth often reduce the effectiveness of maxillary expansion alone in achieving the desired skeletal outcomes. Consequently, rapid maxillary expansion in adults often requires a multidisciplinary approach combining maxillofacial surgery and orthodontic procedures [6,7]. Surgically assisted rapid palatal expansion (SARPE) is a procedure aimed at achieving skeletal maxillary expansion in patients with completed skeletal growth. It involves surgically releasing the resistance areas of the maxilla and expanding it with the help of orthodontic appliances [8]. These resistance areas include anterior support (the piriform aperture), lateral support (the zygomatic buttress), posterior support (the pterygomaxillary junction), and median support (the midpalatal suture) [8,9,10].

In SARPE, various osteotomy techniques have been proposed based on regions identified as resistance sites. In 1938, the midpalatal suture was suggested as the primary resistance point in maxillary expansion, leading to the introduction of the midpalatal osteotomy [11]. Following this approach, different osteotomy techniques have been developed. Some researchers have advocated that a minimally invasive osteotomy limited to the midpalatal suture would be sufficient for maxillary expansion, while others have argued that bilateral lateral osteotomy alone could achieve the desired results without the need for a palatal osteotomy [12,13,14]. Additionally, techniques combining both approaches—preserving the pterygomaxillary junction while performing lateral maxillary and midpalatal osteotomies—have also been proposed [15,16]. One commonly debated topic is whether to separate the pterygomaxillary junction [17], as its separation presents certain difficulties, such as the need for general anesthesia and an increased risk of complications due to the neurovascular complex in the region. However, some authors have argued that this junction is one of the primary resistance areas and, when separation is not performed, expansion in the premolar region is reduced [18,19,20]. Therefore, understanding stress distribution and resistance points in the maxilla and surrounding anatomical structures during SARPE is essential for determining the appropriate osteotomy technique.

Finite element analysis (FEA) is a computational method used to simulate complex geometries and analyze mechanical properties such as stress, strain, and deformation [21]. By modeling structures under various conditions, FEA predicts their response to external forces [22]. Its key advantage lies in enabling simulations of treatment strategies without requiring human or animal studies, thus offering repeatability and efficiency [23]. FEA facilitates the simulation of teeth, alveolar bone, the periodontal ligament (PDL), and craniofacial structures. When material properties are defined to reflect the oral environment, orthodontic forces and techniques can be modeled, allowing theoretical calculations of stress and displacement in the craniofacial system [24]. Stress types evaluated by FEA include maximum principal (Pmax) stress (tension), minimum principal (Pmin) stress (compression), and von Mises stress. While von Mises stress is primarily used to determine the elastic limit in ductile materials, principal stresses are more suitable for analyzing tension and compression in brittle materials [25].

In this study, the effects of various osteotomy techniques and their combinations were evaluated on seven different models using a tooth-borne maxillary expansion appliance and the FEA method. The primary objective was to comprehensively analyze three types of stress (von Mises, Pmax, and Pmin stress values) on the maxilla and surrounding anatomical structures, as well as the displacement amounts at specific anatomical points in the bone and teeth. Although studies comparing the effects of different osteotomy techniques on maxillary expansion have been reported in the literature, research examining these stress types and the displacement effects on teeth and bone points simultaneously is significantly limited. Therefore, this study aimed to contribute to the existing literature in this field.

## 2. Materials and Methods

### 2.1. Design of the Facial Skeleton Models for Finite Element Analysis

In this study, SARPE was simulated using seven different osteotomy techniques. The stress values, distributions, and areas of concentration created in the bone and anchorage units by the tooth-borne appliance were examined in all models. Additionally, the instantaneous displacements in the teeth and bone resulting from the applied forces were evaluated. The research was conducted via static linear analyses using three-dimensional (3D) finite element stress analyses.

The organization of a 3D mesh structure and its conversion into a mathematically appropriate solid mesh structure, the creation of 3D FEA models, and the finite element stress analysis were performed on HP workstations (HP Development Company, Reading, UK) with a 2.40 GHz processor speed, Intel Xeon E-2286 processors, and 64 GB ECC memory. A fully dentate maxilla and a craniofacial model were 3D generated using tomography data obtained from an open-source repository (Visible Human Project, US National Library of Medicine, https://www.nlm.nih.gov/research/visible/visible_human.html, accessed on 19 July 2023) and organized based on anatomical atlas references. The tomography data were reconstructed with a slice thickness of 0.1 mm. The obtained tomography data in DICOM (.dcm) format were transferred to 3DSlicer software (Rhinoceros 4.0; Seattle, WA, USA), where they were processed based on relevant Hounsfield values. The data were converted into a 3D model through segmentation and then exported in stl format. Reverse engineering and 3D CAD activities were performed using ANSYS SpaceClaim software (Ansys Inc., Canonsburg, PA, USA). The process of adapting solid models to the analysis environment and generating the optimized mesh grid was carried out using ANSYS Workbench software (Ansys Inc., Canonsburg, PA, USA).

The data imported into ANSYS SpaceClaim software were used to model cortical and trabecular bone, the PDL, and the maxillary teeth. The cortical bone thickness was set to 2 mm, and the PDLs were modeled with a thickness of 0.2 mm—using the external surface of the teeth as a reference. In all models, a stainless-steel, tooth-borne appliance with a diameter of 1.8 mm and a length of 8 mm, featuring a full turn of 1 mm, was used and modeled. The tooth-borne appliance was positioned to connect to the first premolar and first molar in all models. The geometric models were created and finalized using ANSYS SpaceClaim software. Following the completion of the modeling process, the geometries were imported into ANSYS Workbench, where they were mathematically defined and prepared for FEA. The solution of the finite element models was performed using the LS-DYNA solver (LSTC, Livermore, CA, USA). The total number of nodes and elements for the models is provided in Table 1. All materials were assumed to be homogeneous, isotropic, and linear elastic. Material properties used in the analysis are shown in Table 2.

### 2.2. Types of Osteotomies Used on the Models

**Figure 1:**
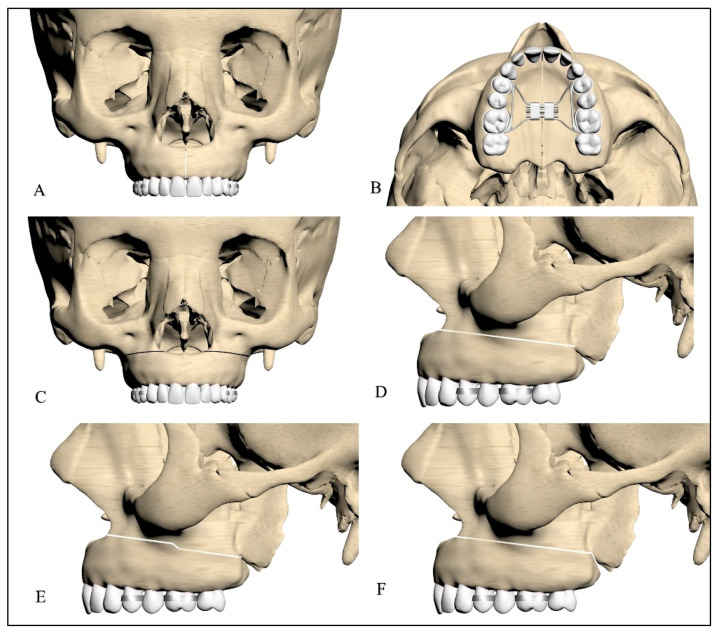
(**A**) Midpalatal osteotomy frontal view; (**B**) midpalatal osteotomy horizontal view; (**C**) lateral osteotomy frontal view; (**D**) lateral osteotomy sagittal view; (**E**) lateral osteotomy with a step; (**F**) lateral osteotomy with pterygomaxillary junction separation.

For this study, 1 mm osteotomies were used on all models. To compare osteotomy techniques, a tooth-borne expansion appliance was used. The models used in the study are presented in Table 3. The designs of the osteotomy techniques are shown in Figure 1.

### 2.3. Loading and Boundary Conditions

For all models, an activation force was applied using the tooth-borne appliance to simulate a total displacement of 1 mm, with 0.5 mm to the right and 0.5 mm to the left. The models were fixed by restricting all degrees of freedom at the nodes in the foramen magnum region, preventing movement in all three axes. The X-axis represents the transverse (buccal–lingual) direction, the Y-axis represents the sagittal (anterior–posterior) direction, and the Z-axis represents the vertical direction. The analysis results, including the von Mises stress values, Pmax, Pmin, and displacement amounts in the three dimensions, were visualized using a color scale. Positive values represent movement in the direction of the arrow, while negative values represent movement opposite to the direction of the arrow. The principal stress results were evaluated based on their absolute values. The stress values were calculated in megapascals (MPa), while the displacement findings were measured in millimeters (mm).

## 3. Results

### 3.1. Amount of Displacement

**Figure 2:**
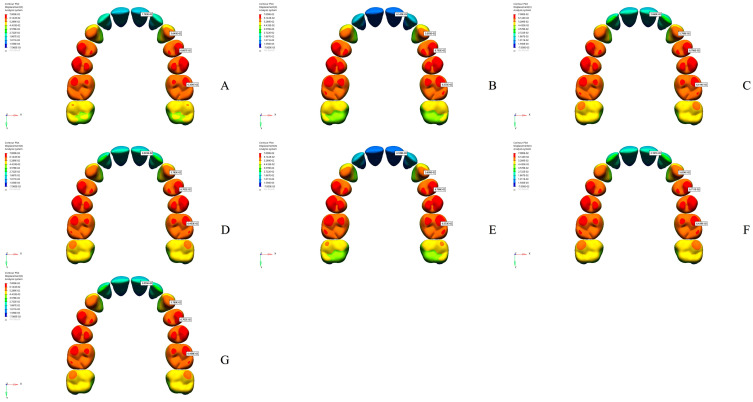
Findings related to tooth displacement along the X-axis (in millimeters). (**A**) Model 1; (**B**) Model 2; (**C**) Model 3; (**D**) Model 4; (**E**) Model 5; (**F**) Model 6; (**G**) Model 7.

**Figure 3:**
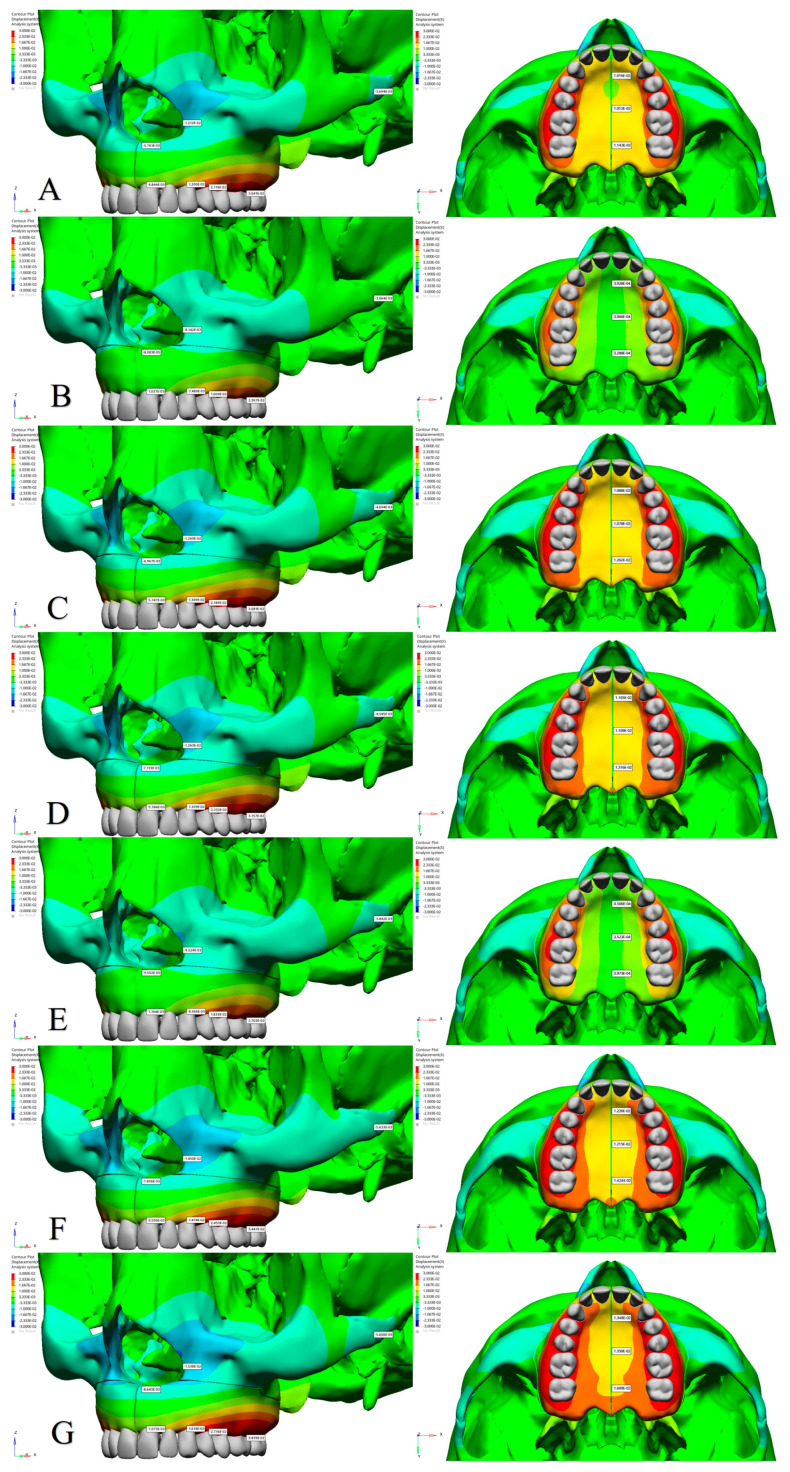
Bone displacement findings along the X-axis (in millimeters). (**A**) Model 1; (**B**) Model 2; (**C**) Model 3; (**D**) Model 4; (**E**) Model 5; (**F**) Model 6; (**G**) Model 7.

The effects of different osteotomy techniques on tooth displacement and anatomical points in the maxillofacial region were evaluated across various models. When evaluating lateral osteotomy effects (Model 1 vs. Model 3), the displacement of the teeth along the X-axis was found to be similar (Figure 2 and Table 4). However, the alveolar bone margin of the central incisor exhibited slightly greater displacement in Model 3, whereas the alveolar bone margins of the other teeth and the intermaxillary suture showed comparable results (Figure 3 and Table 5). In Model 3, along the Y-axis, an increase in posterior displacement was noted only in the alveolar bone margins of the first premolar, while the displacement of other teeth and the intermaxillary suture remained comparable to Model 1 (Figure S1). On the Z-axis, the alveolar bone margins of the central incisor and canine exhibited greater inferior displacement, whereas the first premolar and first molar showed superior displacement in Model 3 (Table 5 and Figure S2).

The application of a midpalatal osteotomy (Model 2 vs. Model 3) resulted in more pronounced lateral displacement of the central incisor and alveolar bone margins along the X-axis and significant lateral movement of the intermaxillary suture in Model 3 (Table 4 and Table 5). Additionally, the nasal base and lateral nasal wall exhibited increased displacement in the medial direction (Figure 3). In Model 3, along the Y-axis, posterior displacement of the alveolar bone margins and the intermaxillary suture was more significant than in Model 2 (Figure S1). On the Z-axis, the alveolar bone margin of the central incisor demonstrated more substantial inferior displacement, while the first premolar showed enhanced superior displacement in Model 3 (Table 5 and Figure S2).

To assess the effects of a stepped lateral osteotomy (Model 6 vs. Model 7), comparative analyses revealed similar displacement magnitudes along the X- and Z-axes across all teeth (Figure 2 and Figure S3). However, along the Y-axis, a slight increase in posterior displacement was noted in Model 7 (Table 4 and Figure S4). Additionally, Model 7 showed a slight increase in lateral displacement at the alveolar bone margins and the intermaxillary suture (Figure 3). Along the Z-axis, the alveolar bone edges of the central incisors, canines, and premolars exhibited increased inferior displacement in Model 7, while the molars showed increased superior displacement (Table 5 and Figure S2).

For all of the teeth, an analysis of pterygomaxillary osteotomy effects (Model 3 vs. Model 6) uncovered similar displacement along the X- and Z-axes (Table 4, Figure 2 and Figure S3). However, Model 6 showed increased lateral displacement at the alveolar bone margins and intermaxillary suture (Figure 3 and Table 5). Along the Y-axis, anterior displacement increased for all teeth and the intermaxillary suture in Model 6 (Figures S1 and S4). On the Z-axis, the central incisor’s alveolar margin exhibited greater inferior displacement in Model 6, while other teeth displayed superior displacement and increased inferior displacement in the intermaxillary suture and nasal structures (Table 5 and Figure S2).

### 3.2. Stress Distribution

**Figure 4:**
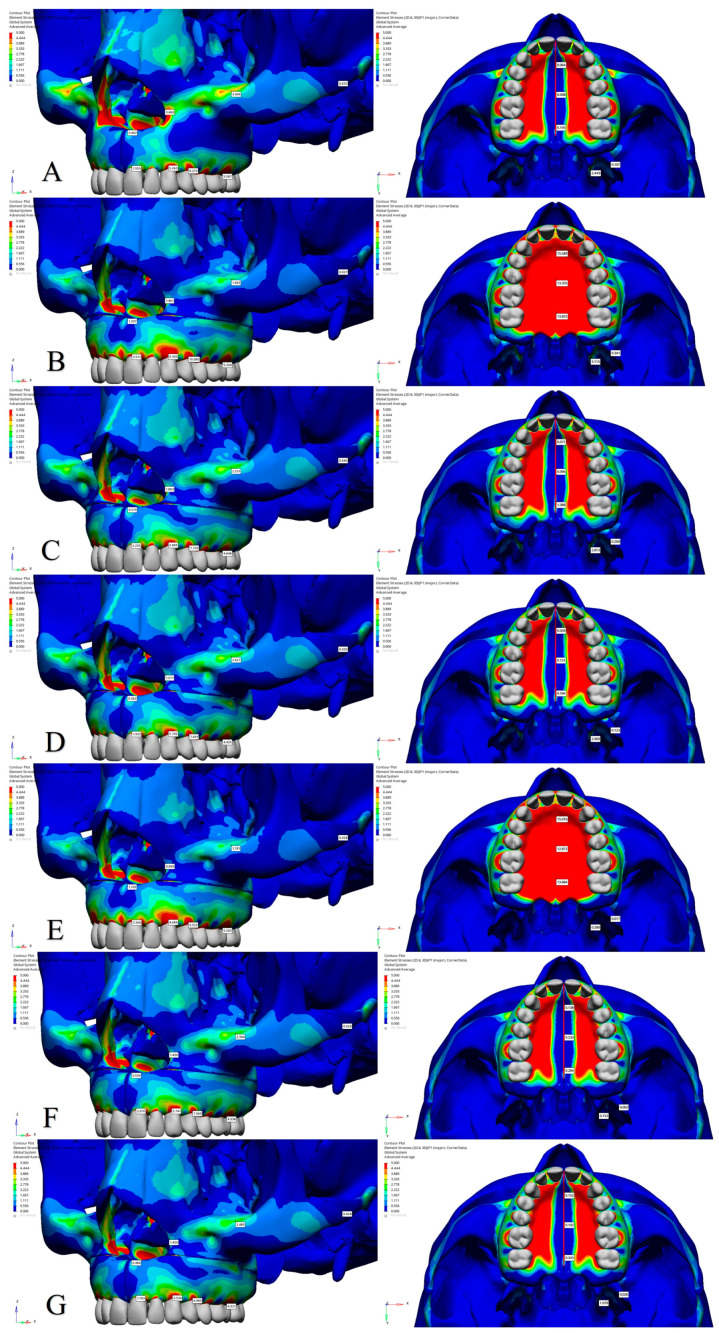
Pmax stress distribution patterns (MPa). (**A**) Model 1; (**B**) Model 2; (**C**) Model 3; (**D**) Model 4; (**E**) Model 5; (**F**) Model 6; (**G**) Model 7.

**Figure 5:**
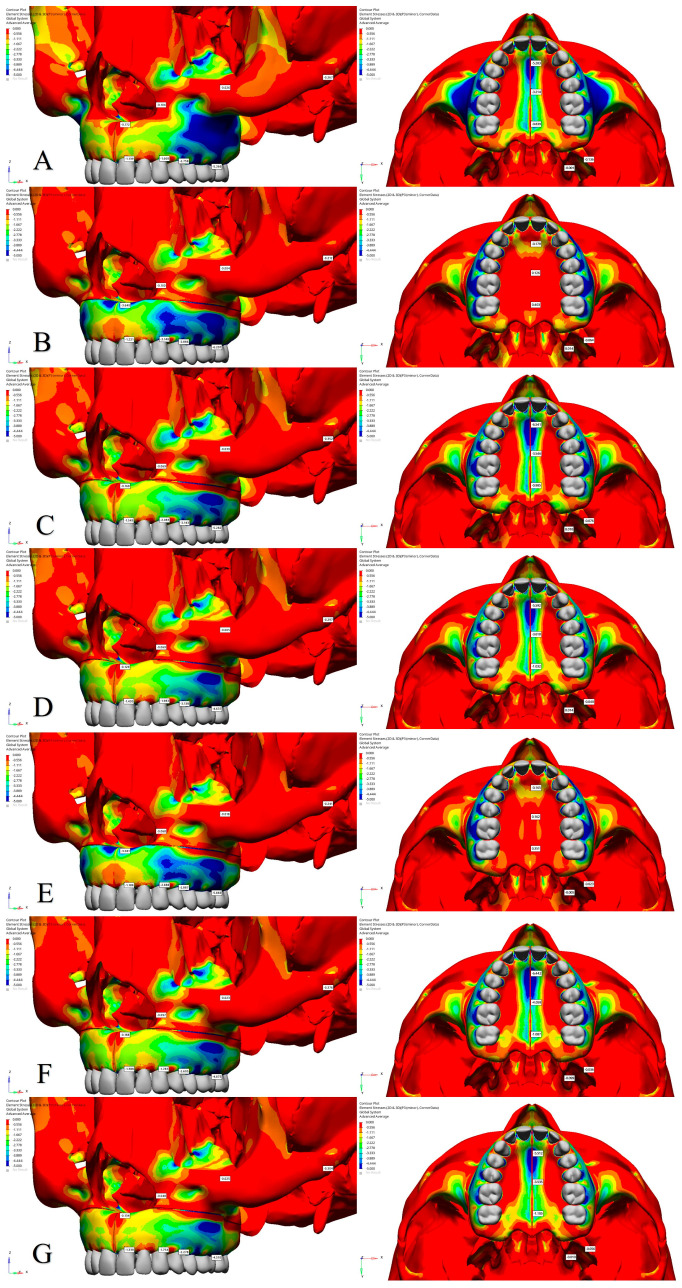
Pmin stress distribution patterns (MPa). (**A**) Model 1; (**B**) Model 2; (**C**) Model 3; (**D**) Model 4; (**E**) Model 5; (**F**) Model 6; (**G**) Model 7.

The effects of midpalatal osteotomy on Pmax, Pmin, and von Mises stress values in Model 3 were analyzed by comparing it with Model 2. The analysis revealed that in Model 3, there was a reduction in von Mises and Pmax stress values at the alveolar bone margins of all teeth (Figure 4 and Table 6). However, in Model 3, an increase in Pmin stress was observed at the alveolar bone margins of the central incisors and first premolars, while a decrease was noted at the margins of canines and first molars (Figure 5). In Model 3, in the anterior and posterior regions of the intermaxillary suture, von Mises and Pmax stress values decreased, whereas Pmin stress showed an increase in both regions. The pterygoid processes exhibited similar stress levels in both models. The application of midpalatal osteotomy in Model 3 was found to substantially reduce stress distribution, particularly in regions such as the intermaxillary suture and the nasal base across all stress types (Table 6).

**Figure 6:**
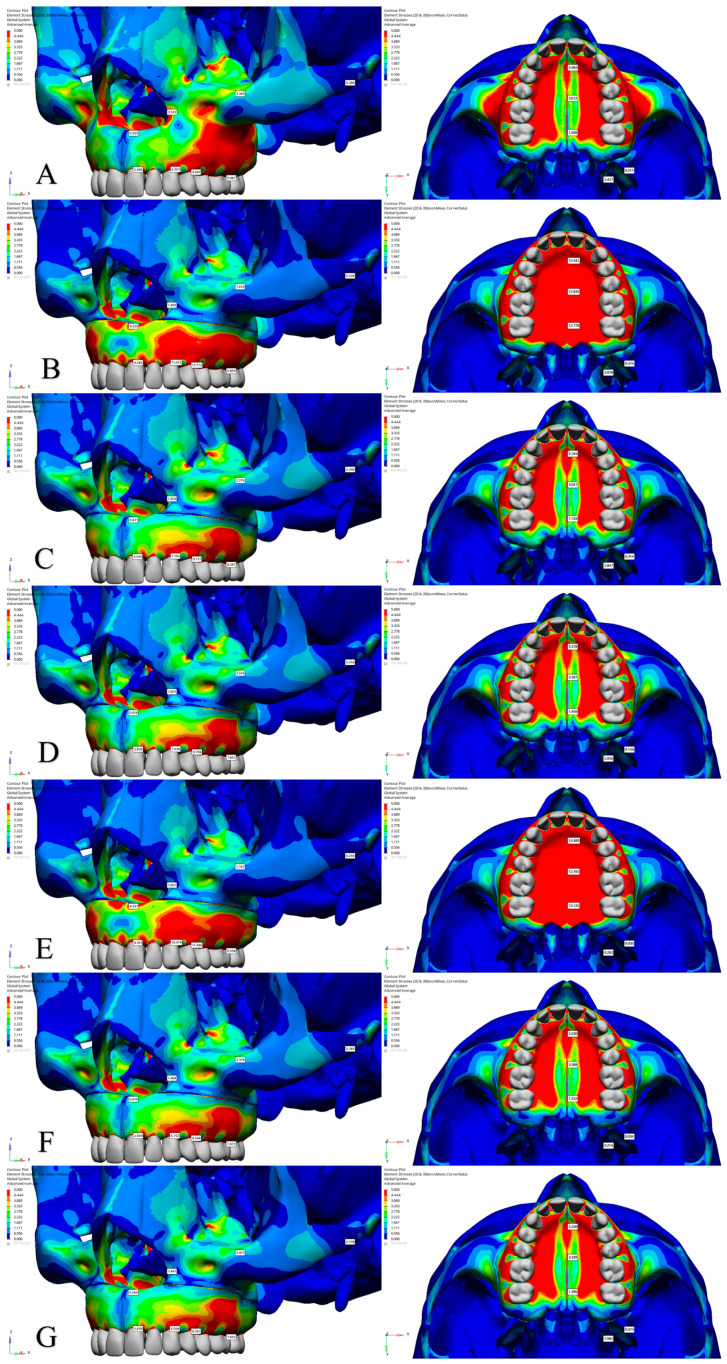
Von Mises stress distribution patterns (MPa). (**A**) Model 1; (**B**) Model 2; (**C**) Model 3; (**D**) Model 4; (**E**) Model 5; (**F**) Model 6; (**G**) Model 7.

The effects of a lateral osteotomy on Pmax, Pmin, and von Mises stress values in Model 3 were analyzed by comparing it with Model 1. The analysis revealed that in Model 3, stress levels at the alveolar bone margins of the central incisor and canine teeth increased across all stress types. The first premolar showed decreased Pmax and von Mises stresses but increased Pmin stress, while the first molar exhibited reduced Pmax and von Mises stresses but with comparable Pmin stress in Model 3. With the addition of lateral osteotomy, stress in the intermaxillary suture increased for all stress types. Additionally, stress at the lateral nasal wall and infraorbital rim decreased for Pmax and von Mises stresses (Figure 4 and Figure 6). Furthermore, the lateral and medial pterygoid processes showed increased Pmax and von Mises stresses and decreased Pmin stress in Model 3 (Table 6).

The effectiveness of a stepped lateral osteotomy was evaluated by comparing Model 3 and Model 4. In Model 4, which represents the application of a stepped lateral osteotomy, a minimal decrease was observed in Pmax, Pmin, and von Mises stress values at the alveolar bone margins of all teeth. In the intermaxillary suture, Pmax and von Mises stress values decreased (Figure 4 and Figure 6). Pmin stress exhibited a slight reduction in the anterior region and a minor increase in the posterior region (Figure 5). Stress levels at the nasal base remained consistent across all types, but Pmax and von Mises stress values decreased at the lateral nasal wall, and Pmin stress values were reduced at the infraorbital rim. In the pterygoid processes, Pmax and von Mises stress values decreased in both the medial and lateral processes, whereas Pmin stress values showed a reduction only in the lateral process (Table 6).

To assess the impact of a pterygomaxillary osteotomy, a comparison was made between Model 3 and Model 6. In Model 6, compared to Model 3, a minimal reduction in stress levels was observed across the alveolar bone margins of all the teeth. In the intermaxillary suture, Pmax stress values increased in the anterior and decreased in the posterior regions (Figure 4). For Pmin and von Mises stress, the intermaxillary suture showed either reductions or similar values (Figure 5 and Figure 6). Minimal decreases were observed in Pmax and von Mises stress values at the lateral nasal wall and infraorbital rim, while Pmin stress values showed a slight increase at the lateral wall and remained consistent at the infraorbital rim. Stress values at the nasal base were generally similar across all three stress types. For the pterygoid processes, reductions in Pmax and von Mises stresses were identified in the lateral pterygoid process. In the medial pterygoid process, Pmax and von Mises stresses decreased, but Pmin stress values increased in Model 6 (Table 6). Overall, a pterygomaxillary osteotomy induced regional variations in stress distribution, contributing to stress reduction in areas such as the alveolar bone margins, intermaxillary suture, and pterygoid processes.

## 4. Discussion

Various osteotomy techniques have been developed for SARPE, and their clinical effects have been demonstrated in numerous studies. However, there is no consensus regarding the most effective and optimal osteotomy technique. While some researchers advocate for the complete release of maxillary structures, others emphasize minimizing osteotomies to reduce postoperative complications and morbidity. This study aimed to evaluate the effects of different osteotomy techniques on stress distribution and displacement in the maxilla to identify the most effective and minimally invasive method.

Zemann et al. reported that a surgical technique combining midpalatal and lateral osteotomies provided more favorable expansion and resulted in less tipping of the teeth compared to models where only a lateral osteotomy was applied [26]. Chung et al. performed the SARPE procedure in all patients, using a tooth-borne expansion appliance. The authors evaluated dental changes by analyzing pre- and post-surgical dental models. They reported a slight mesiobuccal rotation and significant buccal tipping in the first premolar and first molar teeth following the SARPE procedure [27]. Furthermore, Ferraro-Bezerra et al. used cone beam computed tomography (CBCT) in a clinical trial to evaluate patients with and without pterygomaxillary osteotomy. The group that underwent osteotomy exhibited greater skeletal expansion in the posterior region, whereas the non-osteotomy group displayed more dental tipping [28]. Sygouros et al. conducted a similar study and reported consistent findings, with more pronounced buccal alveolar bending and dental tipping in the non-pterygomaxillary osteotomy group compared to the pterygomaxillary osteotomy group [20]. Consistent with the findings of Chung et al. [27] and Zemann et al. [26], our study showed varying degrees of buccal tipping in the first premolar and first molar teeth across all models. Notably, Model 2 and Model 5, in which no midpalatal osteotomy was performed, exhibited the greatest dental tipping in the supporting teeth. Furthermore, an anterior rotation of the buccal cusp was detected in all premolars, with the most pronounced effect seen in Model 5. This outcome can be attributed to the increased bone resistance observed in patients who did not receive a midpalatal osteotomy and to the forces exerted on the supporting teeth during appliance activation, which, instead of contributing to skeletal expansion, led to increased dental tipping. Moreover, in our study, buccal tipping values in the premolar region were similar between models with and without pterygomaxillary osteotomy, while skeletal expansion was increased in models with pterygomaxillary osteotomy. On the other hand, in the molar region, pterygomaxillary osteotomy not only enhanced skeletal expansion but also reduced buccal tipping in the molar teeth.

In the literature, bone–tooth-borne and bone-borne appliances used with the rapid maxillary expansion (RME) method have been compared using FEA. Bone-borne appliances were reported to generate more stress than bone–tooth-borne appliances [29]. However, studies comparing the effects of different appliances on SARPE have demonstrated that bone-borne appliances produce less stress and result in greater skeletal effects than tooth-borne appliances, which tend to cause more dentoalveolar effects [30]. In our study, tooth-borne appliances were used for maxillary expansion in all models, aiming to evaluate the dental and skeletal effects of different osteotomies while keeping the appliance’s effect constant. Although dental effects were observed in all teeth, the least dentoalveolar effect was noted in Model 6.

De Assis et al. have highlighted that the effects of SARPE extend beyond the maxillary dental arch, influencing the nasal septum, nasal base, and lateral nasal walls due to lateral maxillary movement and the involvement of adjacent structures during appliance activation. [31] Pavlin et al. reported that the lateral rotation of maxillary segments during maxillary expansion propagates through surrounding anatomical structures, resulting in increased stresses on nasal structures. These authors emphasized that these transmitted stresses contribute to the medial displacement of the lateral nasal walls. Additionally, Pavlin et al. posited that the pain and sensation of pressure experienced by patients undergoing maxillary expansion may be directly associated with this biomechanism [32]. Consistent with these findings, this study observed medial displacement along the X-axis in the lateral nasal wall across all models. However, minimal medial displacement was noted in Models 2 and 5, which lacked a midpalatal osteotomy. This limited displacement may result from restricted expansion and rotational effects transmitted to adjacent bones without a midpalatal osteotomy. Moreover, Model 1, which did not include a lateral osteotomy, demonstrated approximately double the Pmax and von Mises stress values in the lateral nasal wall compared to models with a lateral osteotomy. This outcome suggests that lateral osteotomy plays a critical role in mitigating rotational forces transmitted from the maxilla to adjacent anatomical structures during expansion. Zawislak et al. used FEA to analyze five osteotomy techniques and observed that midpalatal osteotomy markedly decreased stress levels at the nasal base compared to models lacking this procedure [33]. Consistent with their findings, the current study also demonstrated that midpalatal osteotomy significantly decreases stress distribution at the nasal base.

Han et al. reported that increasing the number of osteotomies in SARPE led to greater maxillary expansion, with the addition of a midpalatal osteotomy to lateral and pterygomaxillary osteotomies significantly increasing posterior expansion [34]. Similarly, in this study, when comparing Models 2 and 3, a midpalatal osteotomy significantly increased lateral movement in the transverse plane, particularly at the alveolar bone margins and the intermaxillary suture. These results align with studies emphasizing the critical role of a midpalatal osteotomy in achieving effective maxillary expansion. Zawislak et al. further demonstrated that stress distribution differs based on osteotomy technique, with lateral osteotomies concentrating stress in the maxilla and midpalatal osteotomies reducing stress in the anterior maxilla and hard palate [33]. In this study, a midpalatal osteotomy significantly reduced Pmax, Pmin, and von Mises stresses across the maxilla and hard palate. Moreover, Model 2 showed greater superior displacement of the buccal cusps of the first premolars and molars compared to Model 3, indicating that, in the absence of a midpalatal osteotomy, supporting teeth are more prone to buccal tipping rather than alveolar expansion.

Chen et al. highlighted in their FEA study that the lateral walls of the maxilla may serve as significant resistance regions against expansion. They found that the time required for midpalatal suture separation was similar between models with only a lateral osteotomy and those with a conventional osteotomy, emphasizing that the former effectively weakens craniomaxillofacial resistance and accelerates midpalatal suture separation [35]. In our study, the model without a lateral osteotomy (Model 1) exhibited concentrated stress areas across the midface, particularly in the infraorbital rim and zygomaticomaxillary buttress. The addition of a lateral osteotomy (Model 3) significantly reduced stress in these regions and the posterior maxilla. However, no significant differences in stress or displacement were observed at the intermaxillary suture and surrounding areas, likely due to the application of a midpalatal osteotomy in both models. Furthermore, adding a lateral osteotomy did not result in substantial lateral displacement of the alveolar bone margins or the intermaxillary suture. Despite reduced stress in the maxilla, the lack of lateral movement suggests that displacement in the model with only a lateral osteotomy (Model 2) may primarily result from the bending of the alveolar bone rather than the widening of the intermaxillary suture.

De Assis et al. further reported that a lateral osteotomy with a step resulted in greater maxillary expansion compared to a lateral osteotomy with no step. In contrast, a lateral osteotomy with no step caused more displacement in the supporting teeth rather than the bone itself [31]. In the current study, stepped osteotomy models exhibited minimal increases in lateral displacement at the alveolar margins and intermaxillary suture. Notably, Pmax and von Mises stresses minimally decreased in these models, while Pmin stresses showed no significant differences. Based on these findings, and in contrast to De Assis et al., it can be concluded that the increased lateral movement and reduced stress in the maxilla did not lead to clinically significant differences between the models.

Zawislak et al., in their FEA study, identified the most effective maxillary expansion in models that combined midpalatal and lateral osteotomies, noting that the addition of a pterygomaxillary osteotomy did not produce a significant difference [33]. In contrast, Möhlhenrich et al. observed that models with a pterygomaxillary disjunction demonstrated increased transverse displacement in the posterior intermaxillary suture and more parallel expansion [36]. Similarly, Dalband et al. reported that greater osteotomy extent resulted in increased transverse expansion, underscoring the importance of a pterygomaxillary osteotomy in posterior maxillary widening [37]. Consistent with these studies, our results showed that a pterygomaxillary osteotomy enhanced transverse displacement in the intermaxillary suture, increased lateral displacement at the alveolar bone margins, and promoted inferior displacement of the midpalatal suture. These findings support reports in the literature suggesting that pterygomaxillary osteotomies facilitate more parallel expansion by enhancing maxillary movement.

The disagreement among surgeons regarding the necessity of releasing the pterygoid process during SARPE may be partly due to considerations of the associated risks and benefits. Although pterygomaxillary osteotomy is an essential component of SARPE procedures, it is linked with various potential complications, such as unpredictable fractures involving the pterygoid processes, posterior wall of the maxillary sinus, skull base, and orbit. These fractures may occur due to improper execution of the osteotomy or the transmission of excessive forces during the procedure. [38,39] Hemorrhage is another complication frequently reported during SARPE. Common sources of bleeding after maxillary orthognathic surgery include the terminal branches of the maxillary artery, particularly the descending palatine artery, the posterior superior alveolar artery, and the pterygoid venous plexus. While intraoperative bleeding is rare, it can occur if the pterygoid process is disrupted [10]. For instance, Newhouse et al. reported a case of life-threatening hemorrhage caused by the rupture of the internal carotid artery following a fracture of the pterygoid process after pterygomaxillary osteotomy [40]. Dergin et al. reported a case of tinnitus following SARPE with pterygomaxillary osteotomy. Such complications emphasize the proximity of the surgical field to critical neurovascular structures and underscore the importance of meticulous surgical planning and execution [41].

Nonetheless, forgoing pterygomaxillary osteotomy during SARPE can lead to various complications, primarily due to the failure to adequately release maxillary resistance points. This incomplete release results in the forces generated by the expansion device being transmitted abnormally throughout the craniofacial complex, often leading to unintended outcomes [38]. These forces may cause aberrant fractures that extend to the skull base, orbit, and pterygopalatine fossa, resulting in injuries to critical neurovascular structures [10]. In a case reported by Li et al., insufficient pterygomaxillary osteotomy resulted in severe complications in a 34-year-old woman with transverse maxillary deficiency. The incomplete release of the pterygoid processes caused excessive forces to be transmitted throughout the craniofacial complex, resulting in an aberrant fracture of the left orbital floor. This fracture resulted in retrobulbar hemorrhage, which subsequently caused orbital compartment syndrome and permanent blindness [42]. In addition, Lanigan and Mintz reported a case of cranial base fracture and retrobulbar hemorrhage in a SARPE procedure performed without pterygomaxillary osteotomy [38].

FEA studies in the literature also confirm that pterygomaxillary osteotomy reduces stress in the cranial base. In a study conducted by Nowak et al., it was reported that in a model where midpalatal, lateral, and pterygomaxillary junction osteotomies were performed, stresses in both the cranial skeleton and the maxilla were significantly reduced. Consequently, they emphasized the importance of lateral and pterygomaxillary osteotomies in reducing overall maxillary stress [43]. Similarly, Holberg et al., in their FEA study, reported that a pterygomaxillary junction osteotomy significantly reduced stress in the pterygoid processes, thereby preventing undesired fractures and microfractures in the cranial base [44].

Zandi et al. conducted a short-term controlled clinical trial to evaluate the application of pterygomaxillary osteotomy and found comparable amounts of expansion in the first premolar and molar regions between SARPE treatments with and without pterygomaxillary osteotomy. Additionally, they reported no significant differences in the maxillary expansion between the two groups. Therefore, they concluded that SARPE without pterygomaxillary osteotomy is recommended for treating transverse maxillary deficiencies [45]. Kilic et al., in their retrospective study investigating the dental and skeletal effects of pterygomaxillary osteotomy during SARPE, reported no significant differences in maxillary expansion between groups with and without pterygomaxillary osteotomy. However, they noted that pterygomaxillary osteotomy may have an advantage because the osteotomy group exhibited enhanced skeletal expansion, whereas the group that did not undergo this procedure showed increased dental tipping [46]. Our findings demonstrated that a pterygomaxillary osteotomy reduced stress in the posterior maxilla and intermaxillary suture, particularly at the pterygoid processes. These findings support previous studies suggesting that pterygomaxillary osteotomies mitigate harmful stress in adjacent anatomical structures, including the cranial base. Furthermore, in our study, the group with pterygomaxillary osteotomy exhibited increased lateral displacement values at the alveolar bone margins and intermaxillary suture. In addition, the model with pterygomaxillary osteotomy exhibited a more parallel expansion pattern. Also, greater skeletal displacement was observed in the model with pterygomaxillary osteotomy, whereas the model without the procedure demonstrated more pronounced dental tipping. Moreover, by reducing stresses, the procedure may contribute to increased stability and more durable long-term outcomes.

The biomechanical properties of bone arise from the interactions among its microstructural components, exhibiting both ductile and brittle behavior. Furthermore, the combination of cortical and trabecular structures contributes to the absorption, distribution, and elastic deformation of applied loads, as reported in the literature [47,48,49].

Von Mises stress is derived from principal stresses and is used to predict the onset of deformation in ductile materials. Moreover, it has been reported in the literature as a reliable method for stress analysis in heterogeneous structures such as bone [47]. Additionally, maximum (Pmax) and minimum (Pmin) principal stresses allow for the separate analysis of tensile and compressive stresses, providing a more detailed picture of the behavior of brittle materials. Pmax evaluates tensile stresses, while Pmin assesses compressive stresses, offering a complementary approach for analyzing brittle structures such as bone [50]. Moga et al. demonstrated that von Mises stress yielded consistent results under various loading conditions in both cortical and trabecular bone components, highlighting its suitability for complex biomechanical structures like bone [47]. In our study, the combined use of von Mises stress alongside Pmax and Pmin criteria aimed to analyze the effects of different osteotomy techniques on bone comprehensively. Von Mises stress was employed to account for the heterogeneous nature of bone, while Pmax and Pmin criteria were used to evaluate the effects of tensile and compressive stresses separately. This approach enabled a detailed analysis of the biomechanical behavior of bone under various loading conditions during maxillary expansion.

The ideal SARPE treatment is specific to each patient and depends on the clinician’s experience, the maturity of the sutures, and the amount of expansion needed. Understanding the biomechanical behavior of the maxillofacial region is challenging because of the mechanical complexity of the stomatognathic system and the difficulty of studying the mechanical properties of the human skeleton in vivo. Therefore, FEA is now widely applied in the study of maxillofacial surgery [51].

As in most FEA studies, our study also has certain limitations. In many FEA studies, modeled structures are typically assumed to be isotropic, homogeneous, and linearly elastic. While this simplification facilitates modeling, it may not accurately reflect the anisotropic and heterogeneous nature of bone tissue within the oral environment. This could affect stress distribution and result in discrepancies between simulated and clinical outcomes. In our study, the maxillary bone was represented as a homogeneous, isotropic, and linearly elastic material. This simplification was necessary for the finite element model but may not accurately capture the true variations in bone density and thickness, which could affect stress distribution. However, to enhance accuracy, Poisson’s ratio and Young’s modulus were separately evaluated for teeth, the PDL, cortical bone, and cancellous bone in our models. Moreover, activation forces and material properties were kept constant across models to allow for controlled comparisons. Because our study focused on comparing different treatment protocols, these limitations are not expected to affect our comparative findings notably. The primary objective was to evaluate the relative performance of each protocol under consistent conditions, which remains valid even with these simplifications.

Additionally, our simulations assumed complete separation of bone fragments, whereas, in clinical practice, the periosteum, soft tissues, and other anatomical structures maintain partial structural integrity. The adaptation of these soft tissues and muscles plays a critical role in maintaining the stability of the achieved expansion because surrounding tissues also undergo stress and adaptive changes during the expansion process [52]. Factors such as palatal shape, suture maturity, and bone density variability also influence maxillary biomechanics, making it challenging for a single FEA model to represent every clinical scenario. For instance, increased bone density with age may limit the amount of expansion [53], whereas palatal morphology can influence the direction and distribution of the expansion forces. While these factors were not explicitly modeled in this study, acknowledging their impact is essential for interpreting the findings within a broader clinical context.

Furthermore, our study focused on controlled conditions and did not account for the variability in force direction or dynamic application of expansion forces. In clinical practice, forces are applied incrementally over time with periods of relaxation, which could substantially impact stress distribution and tissue adaptation. Thus, incorporating dynamic simulations in future studies could enhance the accuracy and relevance of the findings.

Finally, the severity of maxillary deficiency could impact outcomes, potentially requiring different surgical approaches or appliance modifications. Such variations underscore the importance of personalized treatment planning and highlight the need for further studies to explore these factors across diverse patient populations.

Despite these limitations, our comparative study provides valuable insights. While numerous FEA studies have investigated the effects of different osteotomy techniques on stress and displacement, no study has, to date, comprehensively compared all proposed techniques and their outcomes. For the treatment of transverse maxillary deficiency with SARPE, it is essential to select the least invasive surgical technique and orthodontic appliance to minimize patient trauma and damage to supporting teeth. Further research that overcomes current limitations is needed to advance understanding and optimize treatment protocols.

## 5. Conclusions

In this study, we found that different osteotomy techniques have a significant effect on the effectiveness of maxillary expansion. The midpalatal osteotomy was the most effective technique, as it increased lateral displacement and reduced stress around the intermaxillary suture. In the absence of a lateral osteotomy, significant stress concentration was observed in the midface, although its effect on lateral displacement was limited. The stepped lateral osteotomy provided minimal benefit in reducing stress and enhancing lateral displacement. The pterygomaxillary osteotomy, however, was particularly effective in reducing stress in the posterior maxilla and contributed to achieving a more parallel maxillary expansion. Our findings suggest that for greater anterior maxillary expansion, midpalatal and lateral osteotomies alone may be sufficient. However, to achieve a more parallel expansion pattern, a combination of midpalatal, lateral, and pterygomaxillary osteotomies may be necessary. Increasing the number of osteotomies reduced stresses and improved lateral displacement, offering clinical practical guidance for optimizing maxillary expansion procedures.

## Figures and Tables

**Table 1:** Number of nodes and elements for the models.

Models	Number of Nodes	Number of Elements
Model 1	1,314,093	5,397,257
Model 2	1,317,030	5,420,606
Model 3	1,322,605	5,420,048
Model 4	1,331,596	5,457,519
Model 5	1,321,567	5,423,891
Model 6	1,326,902	5,446,452
Model 7	1,331,933	5,483,917

**Table 2:** Material properties of bony structures, teeth, periodontal ligament, and tooth-borne appliance.

	Elastic Modulus [MPa]	Poisson Ratio
Cortical bone	13,700	0.30
Cancellous bone	1370	0.30
Teeth	18,600	0.31
Periodontal ligament	69	0.45
Stainless steel (tooth-borne appliance)	200,000	0.30

**Table 3:** Comparison of the study models.

Models	Appliance	Osteotomy Technique
Model 1	Tooth-borne	Midpalatal osteotomy
Model 2	Tooth-borne	Lateral osteotomy
Model 3	Tooth-borne	Midpalatal and lateral osteotomy
Model 4	Tooth-borne	Midpalatal osteotomy and stepped lateral osteotomy
Model 5	Tooth-borne	Lateral osteotomy with pterygomaxillary junction (PMJ) separation
Model 6	Tooth-borne	Midpalatal osteotomy and lateral osteotomy with PMJ separation
Model 7	Tooth-borne	Midpalatal osteotomy and stepped lateral osteotomy with PMJ separation

**Table 4:** The displacement values of teeth in all of the models, presented in millimeters (mm).

Anatomical Structure	Model 1			Model 2			Model 3			Model 4			Model 5			Model 6			Model 7		
	X	Y	Z	X	Y	Z	X	Y	Z	X	Y	Z	X	Y	Z	X	Y	Z	X	Y	Z
**Central Incisor**	0.01783	0.01069	−0.02418	0.004140	0.01296	−0.02368	0.02069	0.01075	−0.02942	0.02049	0.01171	−0.02966	0.004189	0.01010	−0.02201	0.02101	0.007598	−0.02791	0.02055	0.01096	−0.02926
**Canine Cusp**	0.05640	−0.003056	−0.004805	0.05593	−0.002874	−0.005458	0.05799	−0.002047	−0.006200	0.05785	−0.001356	−0.006336	0.05609	−0.005288	−0.004487	0.05824	−0.004553	−0.005334	0.05789	−0.001956	−0.006106
**First Premolar Buccal Tubercle**	0.06667	−0.006613	0.005785	0.06792	−0.007314	0.006767	0.06706	−0.005258	0.005218	0.06702	−0.004589	0.005205	0.06796	−0.009684	0.007267	0.06711	−0.007692	0.005617	0.06702	−0.005173	0.005324
**First Molar Mesiobuccal Tubercle**	0.06354	0.001799	0.006254	0.06535	0.001564	0.008392	0.06414	0.001860	0.007051	0.06403	0.002514	0.007204	0.06553	−0.001047	0.008062	0.06439	−0.0007814	0.006672	0.06408	0.001900	0.007142

**Table 5:** The displacement values of the bone in all models are presented in millimeters (mm) in the table.

Anatomical Structure	Model 1			Model 2			Model 3			Model 4			Model 5			Model 6			Model 7		
	X	Y	Z	X	Y	Z	X	Y	Z	X	Y	Z	X	Y	Z	X	Y	Z	X	Y	Z
Central Incisor Alveolar Ridge	0.004844	0.01022	−0.02146	0.001037	0.007736	−0.01776	0.005747	0.009773	−0.02589	0.005784	0.01057	−0.02672	0.001194	0.007304	−0.01857	0.006556	0.008960	−0.02715	0.007073	0.01232	−0.03200
Canine Alveolar Ridge	0.01200	0.002844	−0.006453	0.007489	0.002740	−0.007208	0.01309	0.003036	−0.007885	0.01329	0.003561	−0.008301	0.008566	0.001735	−0.007164	0.01474	0.001660	−0.007645	0.01619	0.003864	−0.009760
First Premolar Alveolar Ridge	0.02119	0.0001255	0.0009165	0.01609	0.0004928	0.0006400	0.02189	0.0006594	0.001427	0.02232	0.001088	0.001265	0.01833	−0.0008134	0.001361	0.02453	−0.0009506	0.002302	0.02716	0.0008673	0.001753
First Molar Alveolar Ridge	0.03041	0.001940	0.003802	0.02367	0.002042	0.005226	0.03081	0.002126	0.006461	0.03157	0.002663	0.006769	0.02703	0.0009805	0.005824	0.03447	0.0007857	0.006988	0.03839	0.002827	0.008189
Nasal Base	−0.00576	0.008583	−0.02547	−0.00008083	0.002814	−0.01729	−0.006967	0.007483	−0.03049	−0.007113	0.007969	−0.03135	−0.00009502	0.002855	−0.01840	−0.007856	0.007789	−0.03284	−0.008643	0.009494	−0.03773
Lateral Nasal Wall	−0.01212	0.006087	−0.005516	−0.008142	0.003612	−0.004369	−0.01269	0.005879	−0.005802	−0.01263	0.006035	−0.005438	−0.009524	0.004038	−0.004645	−0.01450	0.006482	−0.006141	−0.01538	0.007301	−0.006506
Zygomatic Arch	−0.00369	−0.002437	0.001004	−0.003064	−0.001685	−0.001156	−0.004694	−0.002756	−0.0009965	−0.004545	−0.002727	−0.0006058	−0.003842	−0.001968	−0.001424	−0.005633	−0.003066	−0.001365	−0.005600	−0.003301	−0.0007841
Anterior Intermaxillary Suture	0.01016	0.01007	−0.02927	0.0003928	0.005842	−0.01879	0.01088	0.009330	−0.03408	0.01105	0.01017	−0.03472	0.0004500	0.005123	−0.02068	0.01226	0.008401	−0.03754	0.01348	0.01182	−0.04203
Posterior Intermaxillary Suture	0.01143	0.004844	−0.02417	0.0003288	0.0008733	−0.01570	0.01262	0.004059	−0.03415	0.01316	0.004272	−0.03417	0.0003973	−0.001495	−0.02158	0.01424	0.001146	−0.04353	0.01600	0.004253	−0.04272

**Table 6:** The Pmax, Pmin, and von Mises stress values of bone in all models (presented in megapascals).

Anatomical Structure	Model 1			Model 2			Model 3			Model 4			Model 5			Model 6			Model 7		
	Pmax	Pmin	VonMises	Pmax	Pmin	VonMises	Pmax	Pmin	VonMises	Pmax	Pmin	VonMises	Pmax	Pmin	VonMises	Pmax	Pmin	VonMises	Pmax	Pmin	VonMises
Central Incisor Alveolar Ridge	1.555	−1.139	2.345	3.517	−1.221	4.285	2.235	−1.543	3.235	1.933	−1.430	2.836	3.390	−1.188	4.161	2.070	−1.388	2.939	1.703	−1.138	2.639
Canine Alveolar Ridge	5.263	−1.668	6.307	9.105	−3.148	11.052	5.895	−2.083	7.192	5.792	−1.983	7.036	8.680	−2.888	10.474	5.741	−1.783	6.752	5.534	−1.754	6.508
First Premolar Alveolar Ridge	8.272	−2.758	9.941	10.080	−2.886	11.772	7.720	−3.143	9.717	7.691	−3.138	9.706	9.523	−2.887	11.286	7.060	−2.605	8.546	6.985	−2.278	8.361
First Molar Alveolar Ridge	5.587	−5.366	9.487	5.204	−6.207	9.899	4.646	−5.282	8.287	4.425	−4.637	7.851	5.203	−5.869	9.598	4.338	−4.875	7.673	4.321	−4.510	7.652
Nasal Base	0.402	−0.172	0.509	1.397	−3.640	4.529	0.513	−0.168	0.621	0.562	−0.129	0.619	1.150	−3.581	4.291	0.504	−0.188	0.610	0.484	−0.134	0.566
Lateral Nasal Wall	3.469	−0.104	3.522	1.461	−0.100	1.499	1.863	−0.069	1.929	1.571	−0.069	1.603	0.969	−0.068	1.007	1.420	−0.097	1.468	1.420	−0.048	1.447
Infraorbital Rim	3.404	−0.026	3.381	1.963	−0.004	1.933	2.531	−0.030	2.576	2.627	−0.005	2.616	1.781	−0.018	1.767	2.394	−0.023	2.378	2.485	−0.025	2.471
Zygomatic Arch	0.032	−0.267	0.286	0.027	−0.212	0.228	0.040	−0.352	0.346	0.036	−0.297	0.316	0.028	−0.241	0.256	0.029	−0.376	0.365	0.024	−0.304	0.318
Anterior Intermaxillary Suture	0.064	−5.203	4.983	15.589	−0.179	15.541	0.071	−6.541	5.746	0.059	−5.592	5.378	15.016	−0.165	14.883	0.138	−6.442	5.630	0.152	−5.512	5.250
Posterior Intermaxillary Suture	0.193	−0.839	1.008	13.825	0.403	12.778	1.088	−0.985	1.748	0.594	−1.032	1.469	13.884	0.351	13.125	0.284	−1.087	1.232	0.345	−1.105	1.396
Lateral Pterygoid	0.107	−0.136	0.211	0.341	−0.064	0.374	0.269	−0.076	0.314	0.123	−0.040	0.146	0.017	−0.023	0.035	0.002	−0.036	0.034	0.026	−0.056	0.073
Medial Pterygoid	2.449	−0.001	2.427	3.115	0.014	3.078	2.813	0.010	2.847	2.060	0.014	2.036	0.280	−0.005	0.282	0.152	−0.099	0.214	1.079	−0.010	1.082

## Data Availability

The original contributions presented in this study are included in the article/Supplementary Materials. Further inquiries can be directed to the corresponding author.

## Supplementary Materials

The following supporting information can be downloaded at https://www.mdpi.com/article/10.3390/jcm14020449/s1, Figure S1: Bone displacement findings along the Y-axis (in millimeters); Figure S2: Bone displacement findings along the Z-axis (in millimeters); Figure S3: Findings related to tooth displacement along the Z-axis (in millimeters); Figure S4: Findings related to tooth displacement along the Y-axis (in millimeters).
