# Supplementary material for: Stress and Displacement Dynamics in Surgically Assisted Rapid Maxillary Expansion: A Comprehensive Finite Element Analysis of Various Osteotomy Techniques
Source: J Clin Med. 2025 Jan 12;14(2):449. doi: 10.3390/jcm14020449 (PMC11766247; doi:10.3390/jcm14020449)
Supplement: Supplementary file 1 [file jcm-14-00449-s001.zip › Supplementary Figures.docx]

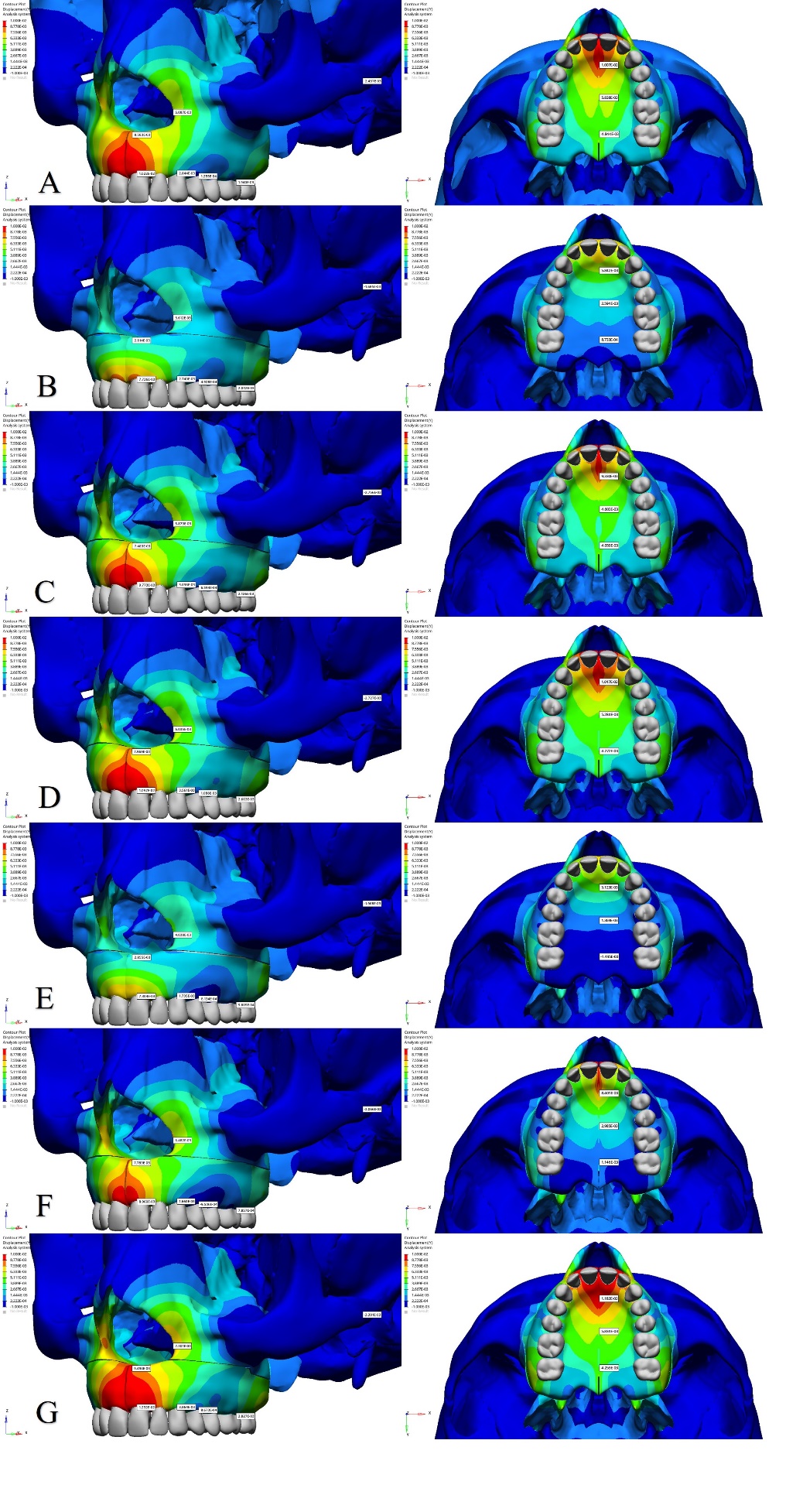


**Supplementary Figure 1**. Bone displacement findings along the Y-axis (in millimeters). **A:** Model 1; **B:** Model 2; **C:** Model 3; **D:** Model 4; **E:** Model 5; **F:** Model 6; **G:** Model 7.


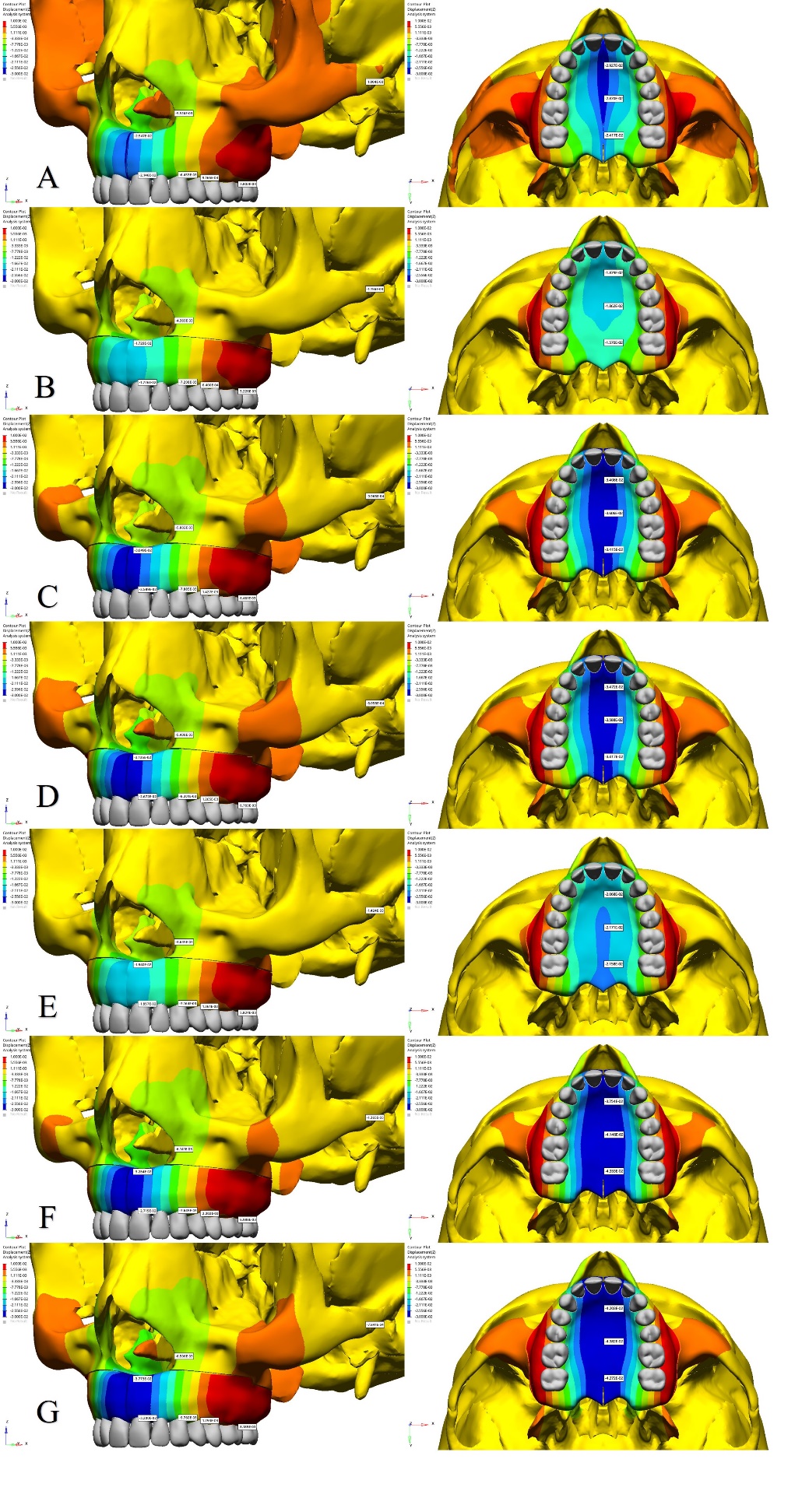


**Supplementary Figure 2**. Bone displacement findings along the Z-axis (in millimeters). **A:** Model 1; **B:** Model 2; **C:** Model 3; **D:** Model 4; **E:** Model 5; **F:** Model 6; **G:** Model 7.


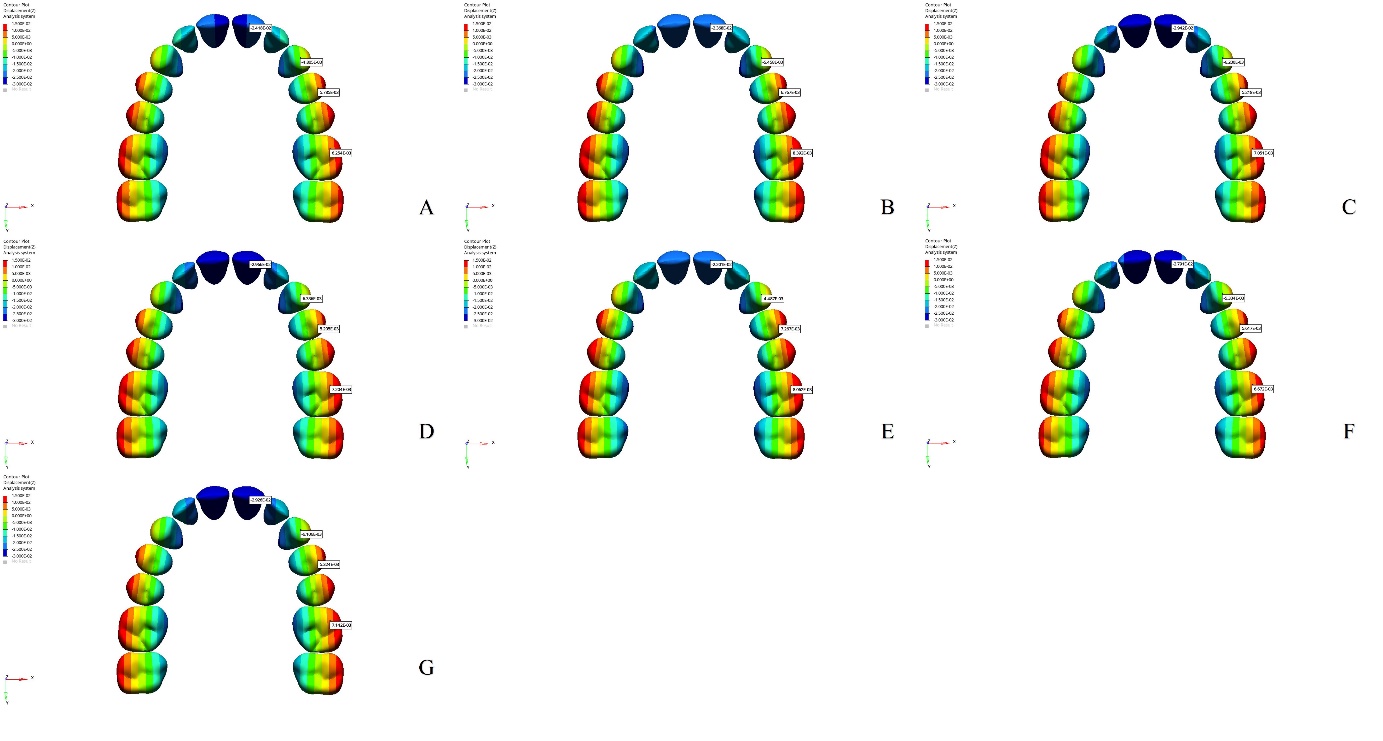


**Supplementary Figure 3**. Findings related to tooth displacement along the Z-axis (in millimeters). **A:** Model 1; **B:** Model 2; **C:** Model 3; **D:** Model 4; **E:** Model 5; **F:** Model 6; **G:** Model 7.


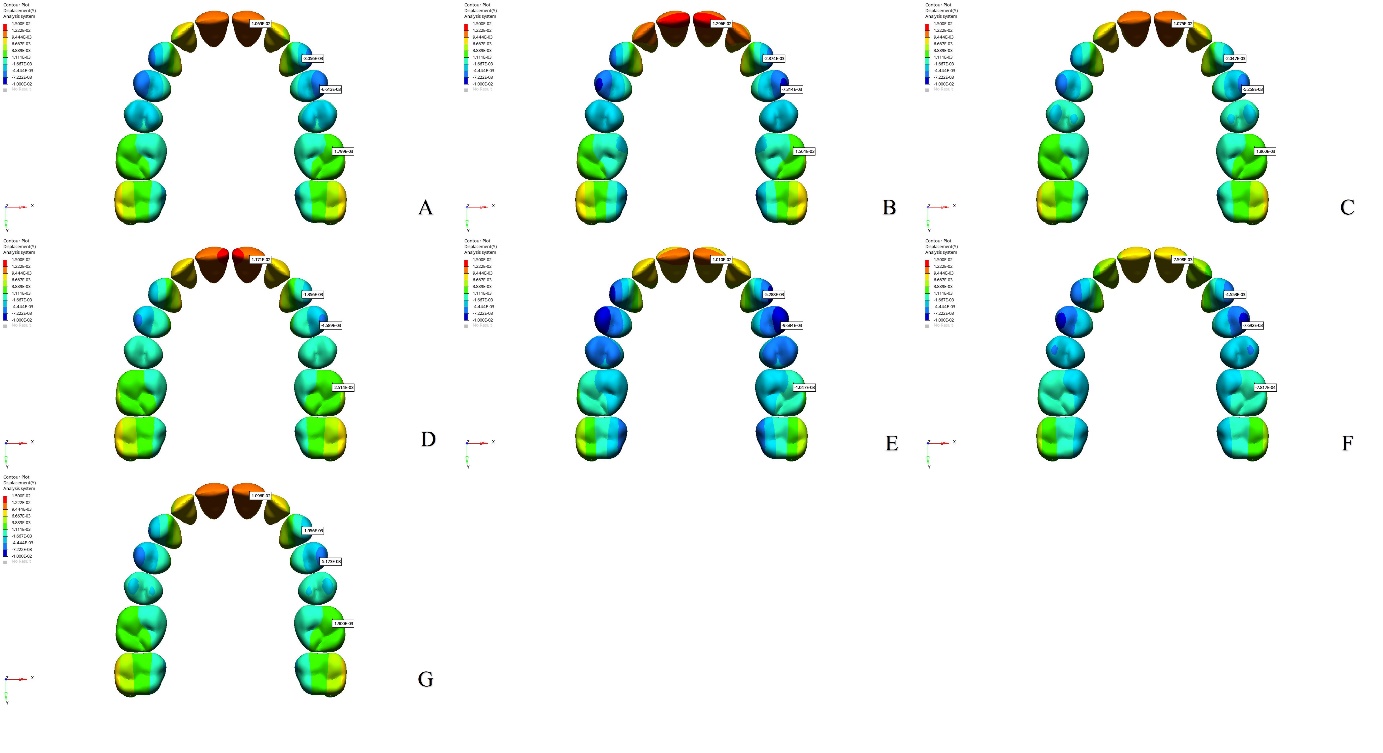


**Supplementary Figure 4**. Findings related to tooth displacement along the Y-axis (in millimeters). **A:** Model 1; **B:** Model 2; **C:** Model 3; **D:** Model 4; **E:** Model 5; **F:** Model 6; **G:** Model 7.
