# Supplementary figures and images for: Stress and Displacement Dynamics in Surgically Assisted Rapid Maxillary Expansion: A Comprehensive Finite Element Analysis of Various Osteotomy Techniques
Source: J Clin Med. 2025 Jan 12;14(2):449. doi: 10.3390/jcm14020449 (PMC11766247; doi:10.3390/jcm14020449)

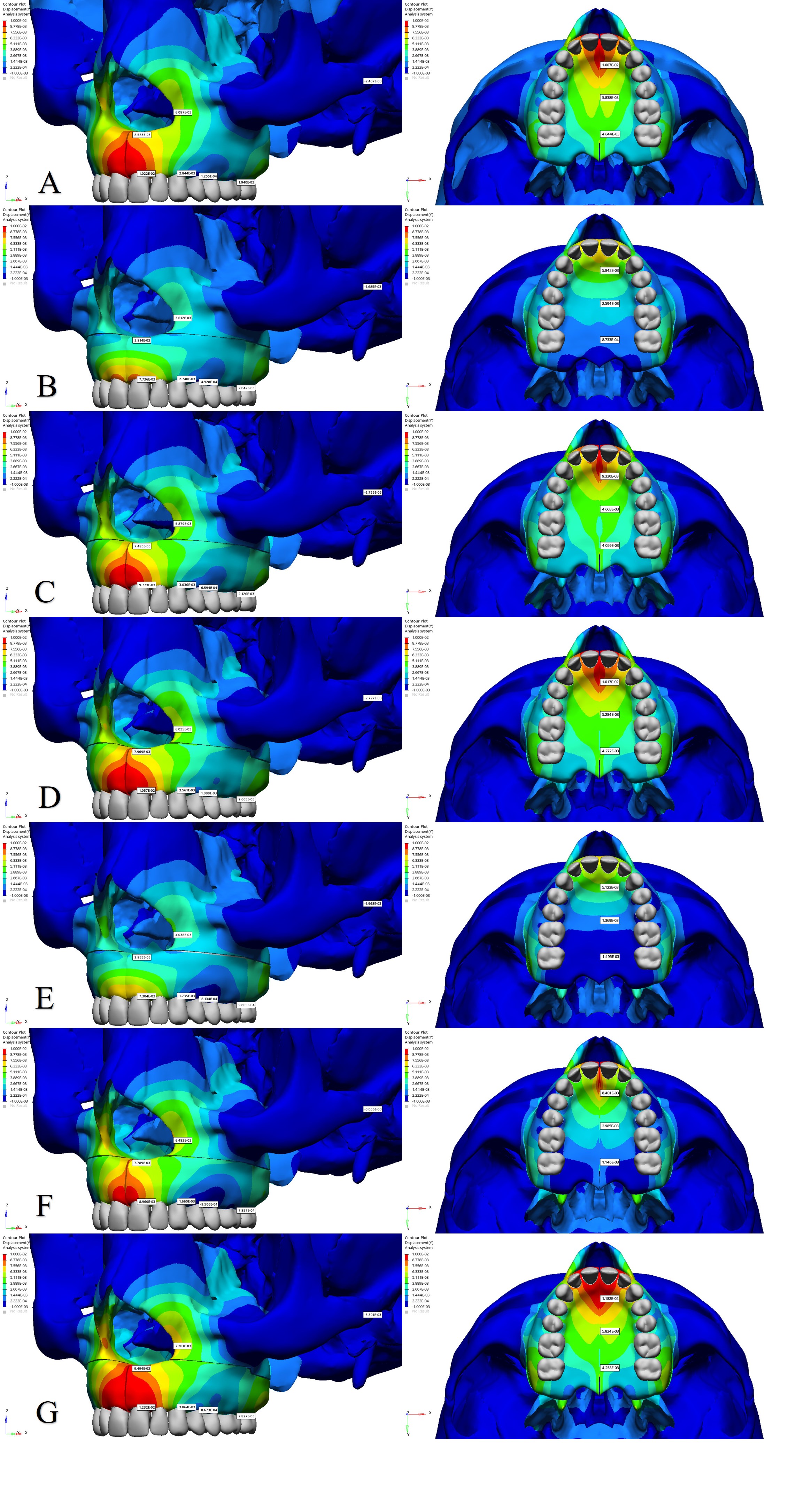

Supplement: Supplementary file 1 [file jcm-14-00449-s001.zip › Figure S1 bone displacement y-axis.jpg]

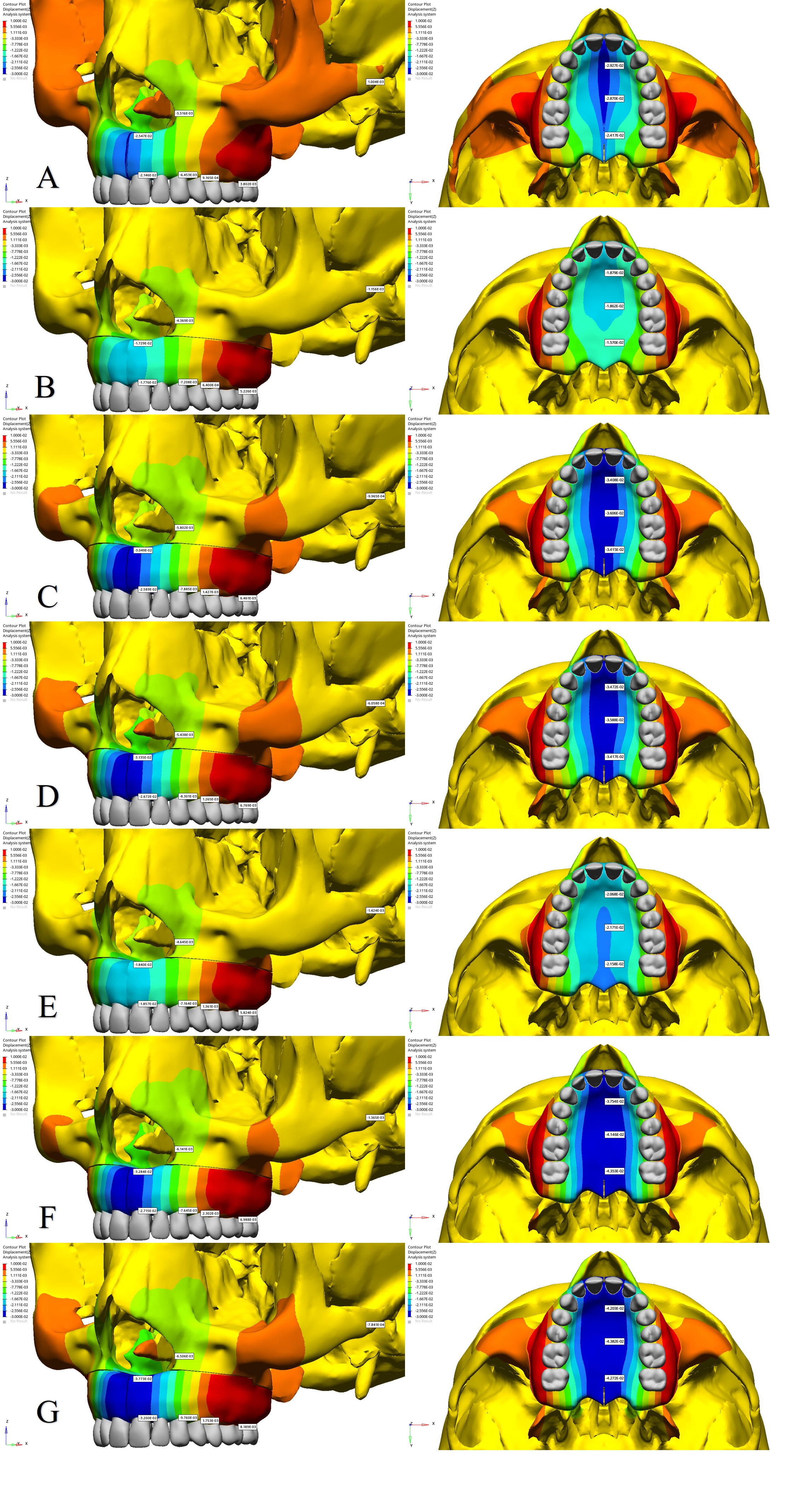

Supplement: Supplementary file 1 [file jcm-14-00449-s001.zip › Figure S2 bone displacement z-axis.jpg]

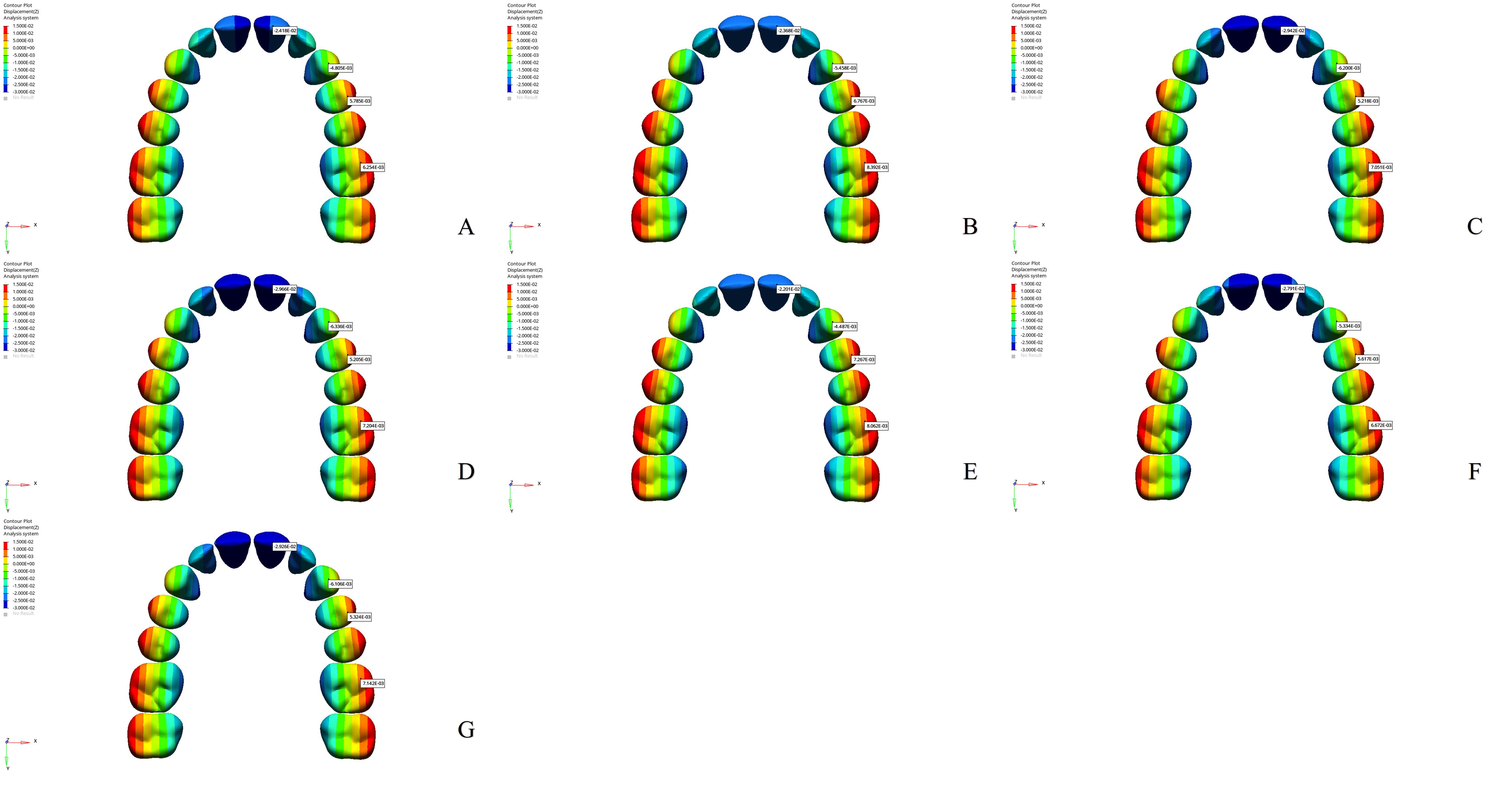

Supplement: Supplementary file 1 [file jcm-14-00449-s001.zip › Figure S3 teeth displacement z-axis.jpg]

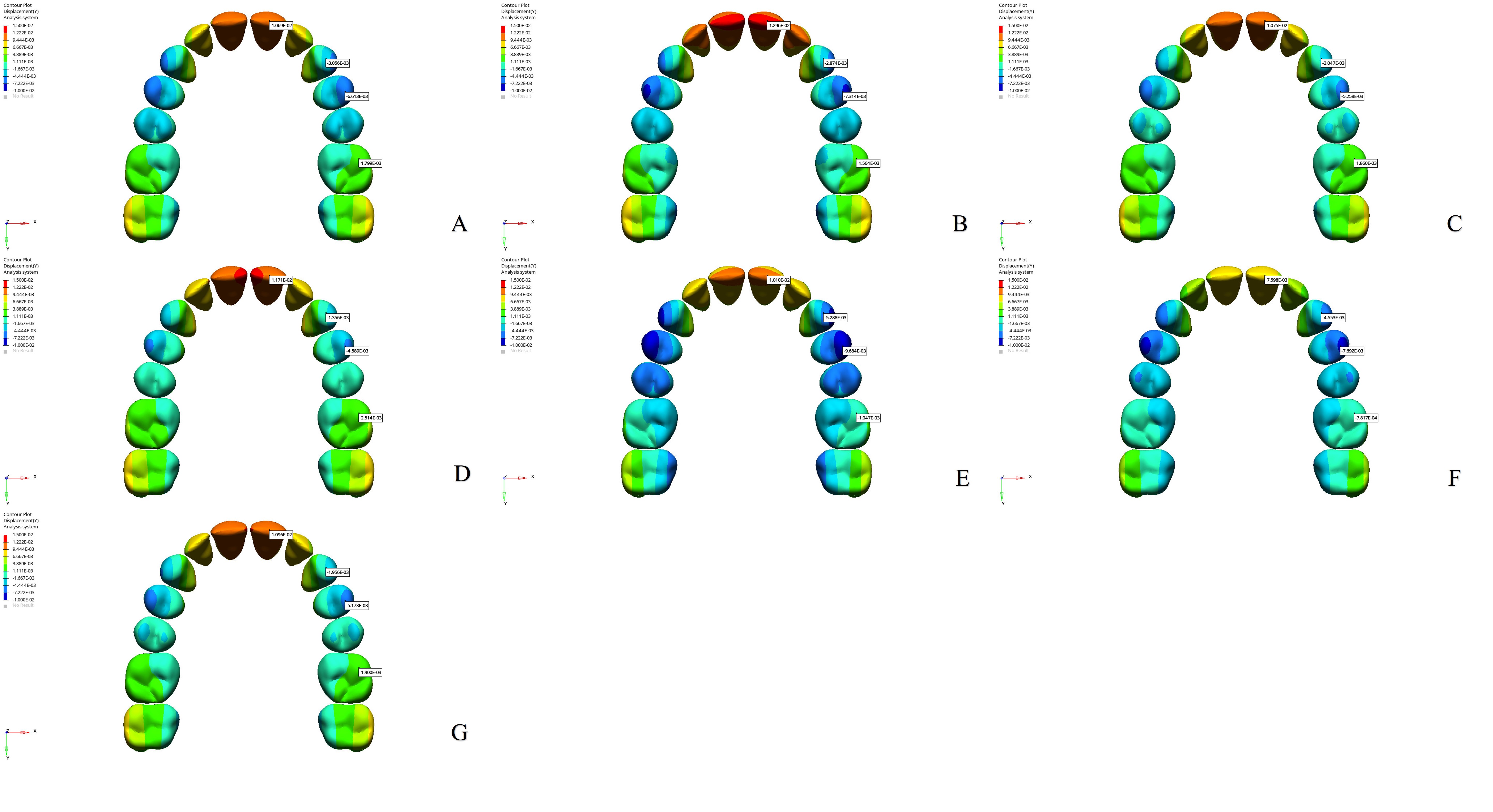

Supplement: Supplementary file 1 [file jcm-14-00449-s001.zip › Figure S4 teeth displacement y-axis.jpg]
